# There or not there? A multidisciplinary review and research agenda on the impact of transparent barriers on human perception, action, and social behavior

**DOI:** 10.3389/fpsyg.2015.01381

**Published:** 2015-09-15

**Authors:** Gesine Marquardt, Emily S. Cross, Alexandra A. de Sousa, Eve Edelstein, Alessandro Farnè, Marcin Leszczynski, Miles Patterson, Susanne Quadflieg

**Affiliations:** ^1^Faculty of Architecture, TU DresdenDresden, Germany; ^2^School of Psychology, Bangor UniversityBangor, UK; ^3^Department of Social and Cultural Psychology, Behavioural Science Institute, Donders Institute for Brain, Cognition and Behaviour, Radboud University NijmegenNijmegen, Netherlands; ^4^Faculty of Psychology, School of Society, Enterprise, and Environment, Bath Spa UniversitySomerset, UK; ^5^College of Architecture, Planning and Landscape Architecture, University of ArizonaTucson, AZ, USA; ^6^ImpAct Team, Lyon Neuroscience Research Center, INSERM U1028, CNRS UMR5292, University Claude Bernard Lyon ILyon, France; ^7^Department of Epileptology, University BonnBonn, Germany; ^8^Department of Psychology, University of Missouri–St. Louis, St. LouisMO, USA; ^9^School of Experimental Psychology, University of BristolBristol, UK; ^10^Division of Psychology, New York University Abu Dhabi, Abu DhabiUAE

**Keywords:** extra-personal space, evidence-based design, translucency, transparency, multisensory integration

## Abstract

Through advances in production and treatment technologies, transparent glass has become an increasingly versatile material and a global hallmark of modern architecture. In the shape of invisible barriers, it defines spaces while simultaneously shaping their lighting, noise, and climate conditions. Despite these unique architectural qualities, little is known regarding the human experience with glass barriers. Is a material that has been described as being simultaneously *there and not there* from an architectural perspective, actually *there* and/or *not there* from perceptual, behavioral, and social points of view? In this article, we review systematic observations and experimental studies that explore the impact of transparent barriers on human cognition and action. In doing so, the importance of empirical and multidisciplinary approaches to inform the use of glass in contemporary architecture is highlighted and key questions for future inquiry are identified.

## Introduction

Everyday experience attests that transparent barriers dominate modern architecture. As surfaces, apertures, windows, or walls, transparent panes of various sizes characterize numerous settings of significance, including airports, offices, schools, hospitals, restaurants, shops, homes, and exhibitions – to name just a few. According to renowned architect Richards (2006), the human fascination with erecting such barriers comes from the fact that they can be *there and not there* at the same time. In other words, while such barriers spatially separate and confine places (including their noise and climate conditions), they simultaneously keep these spaces visually connected. Thus, transparent barriers transmit light in a manner that enables us to see what is beyond them without allowing us to directly approach (or be approached by) what we see. In spite of these unique architectural qualities, the impact of transparent barriers on human cognition and behavior has not yet attracted much scientific attention.

This oversight is surprising given that the optimal architectural use of transparent structures depends not only on esthetic concerns, technological progress, and cost-efficiency, but also on the materials potential to serve setting-specific human needs and goals (cf. Steiner and Veel, 2015). In addressing the latter two issues, this article examines what is currently known, but also what remains to be studied, about human functioning in the presence of transparent barriers. More specifically, this review explores the extent to which barriers that are simultaneously *there and not there* from an architectural perspective, are actually *there* and/or *not there* from perceptual, behavioral, and social points of view. The reader is first introduced to the role of transparent barriers as architectural elements throughout the centuries. Subsequently, systematic observations and experimental studies that have begun to quantify the impact of transparent barriers on human functioning are summarized and discussed. Finally, the practical importance of empirical investigations on transparent barrier use and their real-world consequences is elucidated^1^.

## The Architectural Use of Transparent Barriers

The use of transparent barriers for architectural purposes was originally intertwined with the human ability to produce glass (cf. Miodownik, 2013). Though the first intentional production of glass by humans took place in the form of beads around 3000 BC (Oppenheim et al., 1970), it was the advancement of a new architecture for cathedrals and churches in the Middle Ages (5th–14th century) that stimulated the production of transparent glass panes in Europe (Elkadi, 2006). During that time, glass began to serve as a decorative material that filled wall openings between load-bearing structures such as pillars, allowing daylight to penetrate buildings in an unprecedented manner (Marks, 1993). Although early glass panes varied in color and translucency, they were generally of modest size. To portray large images of Biblical scenes, several small panes would occasionally be joined together by metal frames. The creation of bigger and fully transparent panels, however, required new methods of glass production. Developed in the Baroque era (15th–18th century), these new production methods came at a significant expense, confining the use of generous glass elements mainly to palaces of the nobility and religious buildings (i.e., cathedrals, churches, and cloisters). The architecture of common dwellings, by contrast, involved translucent filters, created from canvas or animal skins.

The modest style of everyday housing quickly changed in the increasingly wealthy European cities at the beginning of the 16th century (Staib, 2008). Especially in Dutch merchants’ houses, load-bearing structures of bricks were increasingly separated from walls that featured large glass openings, enabling passersby to glance at a buildings’ interior. This tendency toward publicizing the private life was further reinforced through the influence of the Calvinists in the 17th century who aimed to demonstrate their pious way of living to God as well as their neighbors (Vera, 1989). Similarly, in 18th century Britain, bow-windows became a popular feature of shops and houses, turning ‘looking in’ as well as ‘looking out’ into common pastimes (Lindsay, 2009). Despite these architectural advances, until the 19th century, glass was primarily used to cover wall openings. Though load-bearing building structures were continuously minimized, stone and clay remained the materials of choice to enclose the interior space. It was not until the development of cast and wrought iron that architects were able to construct magnificent buildings from steel and glass alone. At the end of the 1820s, the first large-scale use of sheet glass appeared in the Parisian Arcades (McQuire, 2003). In 1851, the English architect Joseph Paxton designed and built the Crystal Palace that hosted the Great Exhibition in London (McKean, 1994). The iconic, largely transparent building paved the way for a modern exploration of clear glass in architecture.

In 1914, the German architect and urban planner Bruno Taut constructed a structure from concrete and glass for the Cologne Werkbund Exhibition, known as the Glass Pavilion (Nielsen and Kumarasuriyar, 2014). The pavilion (for a detailed description see Haag Bletter, 1981) was financed by the German glass industry and aimed to illustrate the potential of different types of glass for architecture (Weston, 2004). During the same year, the German writer Paul Scheerbart dedicated an influential monograph to Taut, called *Glass Architecture* (Scheerbart, 1914). In this monograph, the author called for eliminating closed rooms by introducing large scale glass walls, rather than mere windows, in future buildings. In line with his suggestion, many leading European architects produced glass designs in the 1920s (cf. Korn, 1968; McQuire, 2003). Based on this progress, the founder of the Bauhaus, Walther Gropius, concluded that glass architecture had overcome its status as a poetic utopia and had turned into an unconstrained reality (Gropius, 1926). Rapid technological innovations, such as the discovery of crack-preventing laminations and coatings and the development of powerful adhesives that allowed connecting multiple glass panels almost seamlessly, further spurred the widespread use of transparent glass (Staib, 2008; Weller and Vogt, 2012). The architecture critics Johnson and Hitchcock (1932) described the cross-cultural dissemination of glass architecture as a signal of globalized minimalistic and functionalist architectural tendencies.

To date, countries around the world use transparent glass in large scale building projects to demonstrate their progress, modernity, and wealth (Kulterman, 1999; Dawson, 2005). This development is intriguing, considering that in many geographic regions (such as the Gulf States) the use of glass leads to tremendous solar gains in a building’s interior, a circumstance that must be countered with energy-consuming air conditioning (Aboulnaga, 2006; Schittich, 2011). Despite this challenge, architects have continued to design and construct glass buildings worldwide (cf. Wigginton, 1996; Krampen and Schempp, 1999; Bell and Kim, 2009). Supported by the emergence of Computer Aided Design (CAD) software in the very early 21st century, the latest developments in glass architecture defy traditional constructional boundaries. Modern glass buildings can form amorphous structures of curvilinear, topographic design that lack right angles or symmetry, commonly referred to as *Blob architecture* (Lynn, 2009). Pivotal examples of contemporary Blob architecture include the National Centre for the Performing Arts in Bejing^2^ or the Great Glass House in the National Botanic Garden of Wales^3^. Aside from its role in large-scale architectural structures, glass has also penetrated modern domestic architecture, as illustrated by Ludwig Mies van der Rohe’s Farnsworth House^4^ or Philip Johnson’s Glass House^5^.

Despite their global distribution, the use of transparent barriers in contemporary construction seems largely governed by esthetic, financial, legal, structural, and city planning concerns (e.g., Elkadi, 2006; Richards, 2006; Haldimann et al., 2008; Staib, 2008; Arbab and Finley, 2010). The human response to these structures, in contrast, suffers from a lack of consideration and systematic evaluation (cf. Sommer, 1974; Hamilton and Watkins, 2009). Yet, among rare advocates of a psychological approach, the indiscriminate endorsement of transparent architecture has caused skepticism (cf. Widrich, 2015). For instance, as early as in 1963, the architectural critic Colin Rowe and the artist Robert Slutzky distinguished between literal transparency (i.e., the “quality of a substance”, p. 46) and phenomenal transparency (i.e., “an intellectual imperative […] for that which should be easily detected”, p. 45) in order to emphasize that physical and psychological states of transparency could, but would not necessarily have to, co-occur in glass buildings. In further support of their argument, the architectural historian Haag Bletter (1981) directly challenged the notion that transparent structures could somehow promote personal or societal progress. The contemporary architects Vidler (1992) and Colomina (2009), finally, went so far as to argue that transparent barriers could even pose psychological hazards by producing an uncanny loss of privacy. Given these concerns and the assumption that architectural structures are generally meant to benefit people, it must be asked whether and how transparent barriers actually impact human functioning and well-being (cf. Stone and Irvine, 1993). To answer this question in a systematic manner and to ultimately optimize the use of transparent barriers in their manifold manifestations, a thorough, data-driven understanding of the human response to such barriers is needed.

## Transparent Barriers: A Visual Challenge

Before being able to evaluate the consequences of transparent barriers on human behavior and wellbeing, it is of crucial importance to understand how people usually notice their presence.

Although transparent materials are frequently detected on the basis of accidental cracks, traces of adhesives, or dirt in everyday life, humans are surprisingly skilled at seeing transparent structures even in the absence of such opaque markers. However, the visual perception of transparent barriers seems largely guided by perceivers’ experience-based expectations of where to find them (e.g., embedded in window frames, Sayim and Cavanagh, 2011). In addition, a person’s situation-specific monitoring for such panes (maybe resulting from a previous collision) and the detection of visual cues that signal the presence of an invisible structure are of essential importance (Awh et al., 2012). The cues that “give rise to the perception of two surfaces, one of which is seen through the other” (Metelli, 1974a, p. 95) have puzzled artists (e.g., Albers, 1963) and vision scientists (e.g., Helmholtz, 1867) for decades. On the basis of their work, it is now assumed that transparency, unlike other visual features such as shape or color, is detected through a combination of informative cues (Wolfe et al., 2005). Phrased differently, the human visual system must integrate various pieces of information to decipher that something see-through (i.e., non-opaque) is present (Kersten et al., 1992).

**FIGURE 1:**
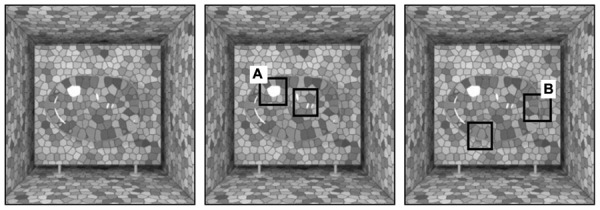
**A computer-generated image that conveys the impression of a smooth, pebble-shaped, transparent blob inside a textured box**. Transparency is conveyed by **(A)** reflections of light (e.g., highlights) as well as by **(B)** refractions of light (e.g., image distortions such as changes in texture and misalignments of borders due to bending of light). Source: From Fleming et al. (2011). Copyright 2011 American Psychological Association; reprinted by permission.

Pivotal cues that facilitate the detection of transparent entities are related to their *reflectance* and *transmittance* properties (Brzezicki, 2013a). Whereas reflectance properties describe the manner in which light is reflected by a material, transmittance properties refer to the way that light passes through it. Interestingly, transparent materials tend to possess specific surface reflectance properties that can give away their presence (Beck and Prazdny, 1981; Blake and Bülthoff, 1990). In particular, glossiness and highlights (see **Figure 1A**) are known to draw perceivers’ attention to transparent structures (Motoyoshi, 2010; Sayim and Cavanagh, 2011). Equally effective at attracting people’s attention to transparency are failures of light transmittance. Although transparent barriers, per definition, transmit the light that falls upon them, the quality of this transmission can vary based on their thickness and unevenness. Specifically, processes of diffusion (i.e., the spreading of light) or refraction (i.e., the bending of light) can result in image distortions that perceivers readily use to infer the presence of a transparent structure (see **Figure 1B**; Fleming et al., 2011). In a closely related manner, the detection of transparent barriers can be aided by their coloring (i.e., due to glazing). Under such conditions, even transparent barriers can absorb some light, thereby producing systematic luminance differences for entities that are seen through them (i.e., affecting their lightness, brightness, and contrast appearance; Masin, 2006; Kingdom, 2011).

**FIGURE 2:**
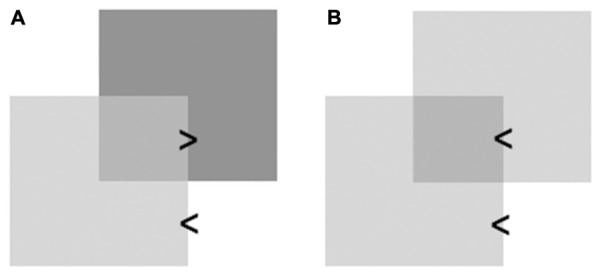
**Illustration of the role of photometric cues in transparency detection. (A)** A set of luminance relations (depicted as light > dark) signaling that the lighter square is seen as transparent and in front of the darker square on the farther surface. **(B)** A set of luminance relations failing to signal which square is seen as transparent and in front of the other.

**FIGURE 3:**
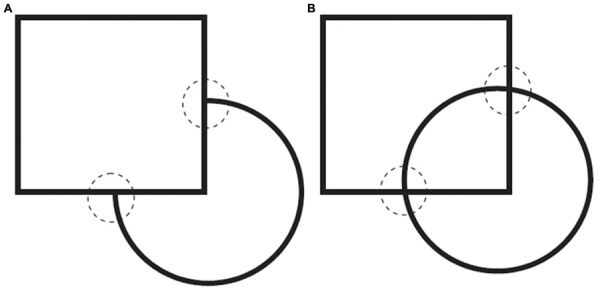
**Illustration of the role of geometric cues in transparency detection. (A)** Opaque square and circle, with T-junctions marked by dashed circles. **(B)** Transparent square and circle, with X-junctions marked by dashed circles. Source: From Hillstrom et al. (2013). Copyright 2013 American Psychological Association; reprinted by permission.

**FIGURE 4:**
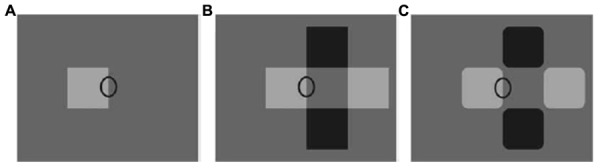
**Illustration of the interplay between border ownership assignment and transparency detection**. The same edge (identified by an identical luminance difference throughout) is perceived as **(A)** part of the contour of the small square, **(B)** part of a black vertical bar that lies underneath a transparent horizontal bar, **(C)** again part of the contour of the small square. Source: From Qiu and von der Heydt (2007). Copyright 2007 Nature Publishing Group; reprinted by permission.

The impact of luminance cues on transparency perception was originally explored by the Italian psychologist Metelli (1970, 1974a,b). Metelli demonstrated that specific luminance differences between neighboring parts of a surface would induce the impression that some parts were seen directly, whereas others were seen through a transparent layer (see **Figure 2A**). In addition to these so-called photometric cues, geometric cues play a pivotal role in transparency detection. The configuration of contour junctions, for instance, frequently signals the relation between two surfaces. Whereas T-junctions typically imply occlusion (**Figure 3A**; Rubin, 2001; Hillstrom et al., 2013), contours aligned in the way of an X-junction (see **Figure 3B**) indicate transparency (Metelli, 1974b; Watanabe and Cavanagh, 1993; Rubin, 2001; Hillstrom et al., 2013). In a similar way, edge assignment can affect transparency perception (Nakayama et al., 1989; Qiu and von der Heydt, 2007). As illustrated in **Figure 4A**, the ellipse marks a border that is considered part of the contour of a small light-gray square. In **Figure 4B**, in contrast, the same visual input (but in a different context) is perceived as the overlapping edge between a horizontal transparent bar lying on top and a darker vertical bar underneath. Yet, when the corners of the light and dark squares are rounded off (**Figure 4C**), the ellipse again appears to mark the border of the small light square. These examples illustrate that numerous visual cues can induce the impression of seeing two surfaces along the same line of sight but at different levels of depths (Beck et al., 1984; Beck and Ivry, 1988; Singh and Anderson, 2002). Phrased differently, these cues enable perceivers to segregate or ‘scission’ visual input into several components, those that constitute a transparent pane and those that constitute opaque entities located behind it (Koffka, 1935; Robilotto et al., 2002; Delogu et al., 2010).

Unfortunately, this segregation process does not always succeed. For example, only specific combinations of luminance relations create the perception of transparency in a unique depth order (Kitaoka, 2005; Koenderink et al., 2010). Alternatively (see **Figure 2B**), perceptions of transparency with an ambiguous depth order due to non-diagnostic luminance relations are possible (creating the impression of so-called bistable transparency; Adelson and Anandan, 1990; Anderson, 1997; Delogu et al., 2010; Fukiage et al., 2014). In addition, if the shape of a transparent pane coincides with the shape of the background, no transparency may be seen (Arnheim, 1971). Such correspondence is not uncommon in architectural settings in which large glass panels fill a person’s entire field of view. Under such conditions, visual information about the transparent structures’ borders and edges remain inaccessible, making their detection particularly difficult (Brzezicki, 2014). Unsurprisingly, undetected transparent barriers can pose a serious health and safety risk. Accidents due to collisions with glass doors or walls have been reported for both children and adults (e.g., Gur et al., 2001; Algaze et al., 2012). Their occurrence has elicited attempts to monitor and standardize the architectural use of glass barriers. In the UK, for instance, health and safety regulations for workplaces require the conspicuous marking of windows and glass doors with warning stickers (United Kingdom Health and Safety Executive, 1992, Regulation 14). In Australia, it has been dictated that glass doors must be made from laminated or toughened glass to reduce the likelihood of serious injuries upon collision (see standards AS 1288 & AS2208; see Kimia et al., 2009; AGGA, 2011). In addition, architects worldwide have been urged to remember that very clear, smooth, faultless, and frameless panes of glass are most likely to result in detection failures (Brzezicki, 2013b).

Although the use of transparent structures in architecture is nowadays expected to be accompanied by a careful assessment of their visibility from different viewpoints and under different lighting conditions (daytime, nighttime, backlighting; Brzezicki, 2014), several important questions regarding the topic remain unanswered. For instance, rather than reflecting an inborn talent, the ability to perceive transparency seems to be actively acquired during the first months of one’s life (Johnson and Aslin, 2000). Exactly how and when the visual system establishes expertise with transparent structures or certain informative cues signaling their presence, however, is an issue of ongoing debate (cf. Otsuka et al., 2006; Kavšek, 2009). Equally unclear is whether frequent exposure to transparent barriers may facilitate a perceiver’s ability to spontaneously detect relevant visual cues. It also remains to be studied whether and to which extent distraction interferes with a person’s capacity to perceive transparent materials, for instance, by impairing the integration of several diagnostic cues. Given that undetected transparent barriers pose the risk of unintentional collisions, finding answers to the above questions is of pivotal relevance for their architectural use.

## Transparent Barriers: A Multi-Sensory Challenge

Not only our sense of sight but also our sense of touch can signal the presence of transparent barriers. Consequently, transparent barrier can confine an individual to a limited physical space (i.e., the person’s sense of touch identifies an impenetrable barrier), while simultaneously providing visual access to a larger space (i.e., the person’s sense of vision fails to identify a barrier). The experience of such divergent sensory information is noteworthy because humans usually create representations of the space closely surrounding their bodies by integrating both tactile and visual information into one multisensory representation (Làdavas and Farnè, 2004; Spence et al., 2004). Compared to mere unisensory analyses, multisensory representations usually result in a more accurate perception of one’s environment (Ernst and Bülthoff, 2004), fostering individuals’ rapid and adaptive responses to their surroundings (Calvert et al., 2004). But what happens when the two sensory systems convey conflicting information, as is commonly the case in the presence of transparent barriers?

**FIGURE 5:**
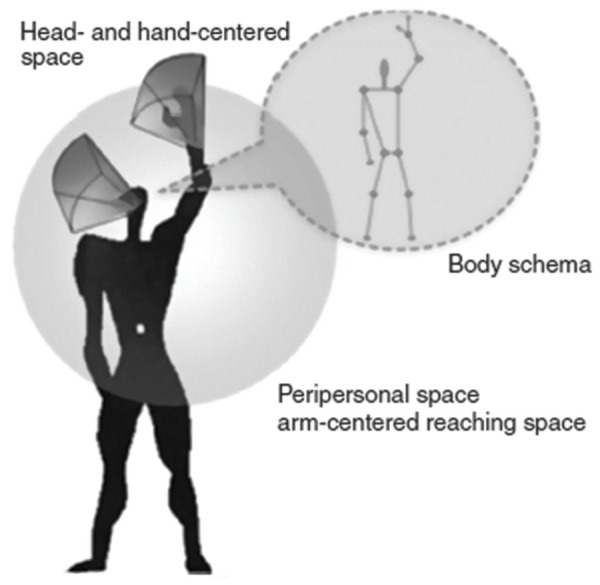
**The peripersonal space, mainly based on the integration of tactile and visual information coming from the body and the space directly surrounding the body, constitutes a privileged interface for the body to interact with nearby objects**. This figure depicts the body schema, head and hand-centered space, and the peripersonal/arm-centered reaching space. Source: From Cardinali et al. (2010). Copyright 2010 Elsevier Publishing Group; reprinted by permission.

Initial evidence indicates that when faced with conflicting information coming from tactile and visual sensors, humans rely more strongly on the latter, especially when the sensory contradictions arise within a person’s peripersonal space (cf. Làdavas et al., 2000). The term peripersonal space denotes the space closely surrounding a person’s body (see **Figure 5**; Previc, 1998; Caggiano et al., 2009; Brozzoli et al., 2014). Conflicting tactile and visual information occurring within this space can lead to intriguing effects. In many species, including humans, avoidance reactions such as head or hand withdrawals are easily triggered by quickly approaching objects that threaten to collide with individuals by entering their peripersonal space (Dunkeld and Bower, 1980; Makin et al., 2009; Serino et al., 2009). Importantly, so-called defensive withdrawal also occurs toward images of objects that rapidly grow in size, even if those images are merely projected on a screen. In other words, under conditions in which objects growing in size seem to approach an individual but actually have no chance of touching him or her, perceivers are still likely to show rapid startle and withdrawal responses toward these objects (King et al., 1992). These findings suggest that, given the appropriate visual input, awareness that one’s body is shielded from events occurring behind a transparent barrier may not suffice to suppress physical arousal and motor reflexes.

Indeed, the ineffectiveness of transparent barriers to interfere with basic reflexes is regularly taken advantage of in medical examinations. For instance, the so-called blink reflex (i.e., a rapid closure of the eyelids to a quickly approaching object such as a thrown ball) is generally tested by separating patients from approaching objects with a piece of plexiglass. Under these circumstances, the transparent barrier not only protects patients from the impact of an impending collision, but also enhances the test’s diagnostic value by shielding them from drafts caused by the motion of the object which may trigger the reflex in a non-visual manner (van Hof-van Duin and Mohn, 1986; Guzetta et al., 2001). Further evidence for a lack of interference of transparent barriers with basic reflexes comes from two behavioral studies exploring the occurrence of so-called visual–tactile interactions (Farnè et al., 2003; Kitagawa and Spence, 2005). The first of the two studies examined a group of *tactile extinction patients* (Farnè et al., 2003). Such patients tend to display an intriguing deficit: a visual sensation experienced in close proximity to one side of their body (e.g., a finger moving down close to their right hand, signaling the potential for touch) inhibits their ability to experience a simultaneously applied tactile sensation to their other side (e.g., a tap applied to the left hand). Crucially, this tactile extinction is less likely to occur the farther the wiggling finger is located from the patient’s hand (i.e., the farther it is located beyond a perceiver’s peripersonal space and the less likely it is to result in physical contact). This pattern of results signals an integrated visual–tactile system coding of peripersonal space in humans. But how is such coding affected by the introduction of a transparent barrier in close proximity to a person’s hand?

**FIGURE 6:**
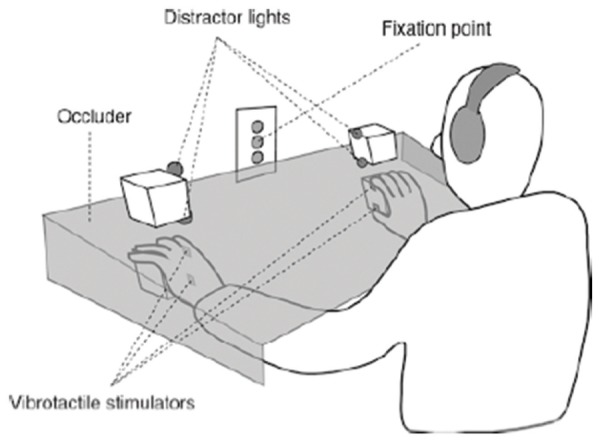
**Schematic illustration of the experimental set-up to study visuo–tactile interactions in healthy adults**. In the transparent barrier condition, the participants’ hands were covered by a transparent Perspex occluder. Source: From Kitagawa and Spence (2005). Copyright 2005 Springer Publishing Group; reprinted by permission.

Under such conditions, the relevant finger simultaneously exists within a person’s peripersonal space (based on its visual properties), but also outside of it (due to being located behind the barrier). Results indicate that even when a wiggling finger is presented behind a transparent barrier, it triggers tactile extinction in these patients, to the same extent as when no barrier is interposed (Farnè et al., 2003). In other words, the patients’ automatic representation of their peripersonal space is unaffected by the presence of the barrier. Thus, the mere knowledge that actual touch is impossible does not modulate the observed extinction effect. A similar observation has been reported for healthy adults (Kitagawa and Spence, 2005). Adopting a so-called cross-modal congruency task, participants received tactile stimulation to either upper or lower portions of the left or right hand. They then had to indicate as quickly and accurately as possible the place of stimulation while simultaneously seeing one of two visual distractor lights near their hands (see **Figure 6**). Again, the presence of a transparent barrier placed between the tactile stimulation and the visual distractors did not impact participants’ performance, indicating that visual–tactile interactions in peripersonal space are unaffected by the presence of transparent barriers.

The above work suggests that people can be fully aware of being protected from collisions or touch by a transparent wall, yet simultaneously show rapid responses to approaching objects or people as if no wall was present. What remains to be studied is whether frequent exposure to and experience with transparent barriers may alter mechanisms of multisensory integration and/or people’s spontaneous responses (cf. Wesslein et al., 2015). The recurrent perception of objects or people behind a transparent barrier may lead to habituation, such that the strength and/or likelihood of reflexive responses toward entities on the other side of a transparent shield may decline over time. If such habituation can be observed, several related questions deserve scientific consideration: (a) How much exposure to transparent barriers does it generally take until habituation is noticeable (i.e., is it a rapid or time-consuming process)? (b) Do some people habituate more or less quickly when shielded by a transparent barrier than others and if so, why? (c) Can habituation effects be transferred across different settings (i.e., would a person that has worked behind a transparent barrier in one setting more quickly habituate when placed behind a transparent barrier in another setting)? Finally, (d) would the habituation of reflexes behind a transparent barrier ultimately compromise a person’s rapid responses in settings that lack such barriers?

## Navigating Transparent Barriers

Further evidence that people are easily inclined to disregard the presence of transparent barriers comes from research on spatial perception and navigation. Navigational research with opaque barriers suggests that humans frequently rely on the presence of walls or fences to orient in space (Acredolo and Boulter, 1984; Herman et al., 1987; Han and Becker, 2013; Buckley et al., 2014). Although such a strategy benefits learning the location of meaningful objects and spatial layouts, it can also distort the representation of spatial information: Distances between entities in different subdivisions of space are judged larger than the same distances between objects located within the same subdivision (Allen, 1981; Maki, 1981, 1982; Newcombe and Liben, 1982). In the case of transparent barriers, however, such spatial overestimation effects seem to be absent (Montello, 2005). The first study on distance estimation, for instance, revealed that preschoolers exaggerated distances between objects separated by both opaque as well as transparent barriers, whereas adults only exaggerated distances across opaque barriers (Kosslyn et al., 1974). A subsequent study demonstrated that neither adults, nor school-aged children overestimated distances between objects separated by transparent barriers (Herman et al., 1987). The absence of a spatial overestimation effect for transparent barriers in viewers familiar with their occurrence indicates that these barriers are not spontaneously used as spatial delineators.

**FIGURE 7:**
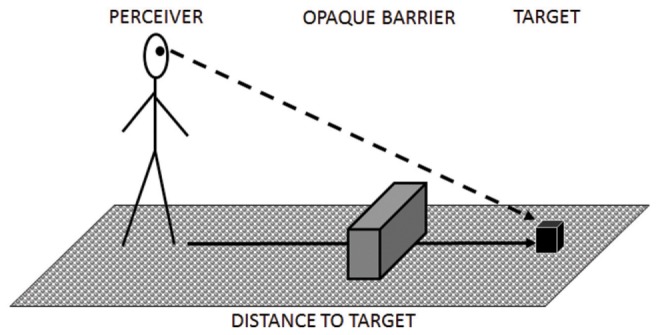
**Schematic illustration of the experimental set-up to study distance-to-target judgments in healthy adults**.

Alternatively, the space-delineating properties of transparent barriers may only arise under specific circumstances. For instance, for short, untraveled distances (i.e., when people merely look at a space, but do not walk around in it), transparent barriers seem to elicit larger (rather than smaller) distance overestimation effects than opaque barriers (Sherman et al., 1979). Moreover, transparent barriers may primarily affect observers’ spatial representations when the barriers are placed between them and a target. Initial research on egocentric distance-to-target judgments suggests that such judgments are more accurate when perceivers are exposed to a continuous, homogenous texture ground surface than to a surface that contains a gap or an opaque barrier (Sinai et al., 1998; He et al., 2004; see **Figure 7**). Whether transparent barriers actually result in accurate or biased judgments is undetermined. An additional issue for further research on transparent barriers is whether distance-to-target judgments tend to be more accurate when taken outdoors (i.e., on a lawn) rather than indoors (i.e., in a hallway or lobby; Lappin et al., 2006). The effect appears to be mediated by the amount and kind of space that is visible beyond an actual target (so-called vista space, Witt et al., 2007). Yet again, due to a scarcity of empirical investigations, the effect of transparent barriers located in vista space on target-to-distance estimates is unclear. In summary, the effects of transparent barriers on spatial representations remain poorly understood. This lack of empirical insight is particularly worrisome as it undermines the ability to predict how people orient themselves in glass environments, a limitation that poses far-reaching health and safety concerns (i.e., in emergency flight situations; Piller and Sebrechts, 2003; Abu-Safieh, 2011).

**FIGURE 8:**
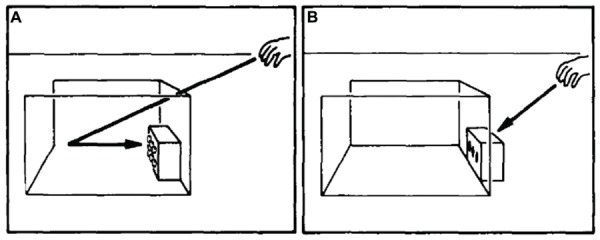
**Experimental set-up to study infants’ toy retrieval in the presence of a transparent barrier**. The arrows signal the required reaching behavior based on whether the transparent barrier **(A)** blocks or **(B)** fails to block direct retrieval. Source: From Diamond and Gilbert (1989). Copyright 1989 Elsevier Publishing Group; reprinted by permission.

These concerns are perpetuated by the observation that humans must actively learn to treat transparent structures as physical barriers. Such learning is most notable in young infants who show a prevalent tendency for reaching or crawling into impermeable transparent surfaces. Butterworth (1977), for instance, tested 9-month-olds search abilities by hiding attractive toys behind opaque or transparent covers. Along similar lines, Diamond and Gilbert (1989) examined 7-11-months-olds’ reach strategies by putting toys in opaque and transparent boxes (see **Figure 8**). In both cases, children younger than 10 months of age tried to directly reach for toys “through” transparent surfaces, even though they readily reached around opaque barriers. Similar observations have also been made regarding young infants’ detour abilities. Lockman (1984), for example, used opaque and transparent barriers to investigate 8-months-olds’ ability to select a path to a goal. This work revealed that, up to 10-months of age, infants displayed significant difficulty in detour ability when obstructed by a transparent barrier. Specifically, they hesitated or refused to select an alternative path to a goal when their current path was blocked ‘merely’ by something transparent (cf. Lockman and Adams, 2001; Noland, 2008). This response pattern does not seem to result from an inability to see the barrier, but rather from a flawed assumption that transparent barriers can be physically penetrated (Johnson and Aslin, 2000; Shinskey and Munakata, 2001). Thus, once this assumption is rectified (e.g., by allowing 9-month-olds to play with transparent covers before testing them) infants systematically remove opaque as well as see-through covers before they reach for a toy of interest (Yates and Bremner, 1988).

These data demonstrate that negotiating transparent materials is developmentally dependent on interacting with them. The term *social ontologies* has been introduced to describe how human capabilities – both in terms of physical and cognitive abilities – are frequently forged through interactions with the human-made material world (Gosden, 2008). Research on barrier crossing provides further evidence that navigating transparent obstacles is an acquired skill. In studies on the topic, young children were asked to step over barriers that varied in height. Subsequently, thresholds for successful barrier crossing (i.e., crossings that do not result in damaging the obstacle) were compared for opaque and transparent barriers. Both 12- and 18-month-olds were found to successfully cross opaque barriers at larger thresholds than transparent barriers, suggesting that a child’s ability to perform an adequate motor response suffers when faced with transparency (Schmuckler, 1996). Additional research is necessary to determine if the observed difference reflects a reduced ability to use cues of transparency for adequate motor planning or whether colliding with transparent instead of opaque barriers may seem less severe to a naïve perceiver (cf. Schmuckler, 1996). Regardless of the underlying mechanism(s), however, these data signal that humans must actively learn to resist the alleged penetrability of transparent barriers.

**FIGURE 9:**
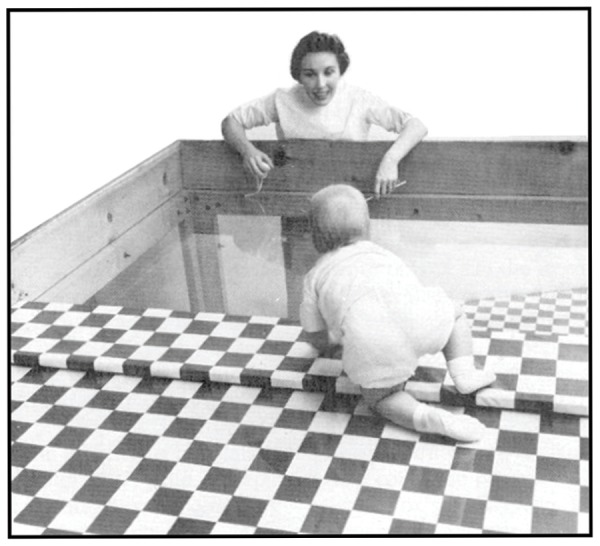
**A mother urging her child from across the deep side of the visual cliff**. Despite a transparent surface covering the cliff, the child hesitates to move forward. Source: From Gibson and Walk (1960). Copyright 1960 Nature Publishing Group; reprinted by permission.

Seminal work on the *visual cliff* further demonstrates the idea that transparent surfaces initially appear immaterial. In this work, the psychologists Gibson and Walk (1960) tested infants’ response to perceived downward depth using a horizontal transparent barrier that covered a cloth with a checkerboard pattern. While the transparent barrier sat directly on the cloth on one side of the apparatus, the cloth was dropped about four feet on its other side. In doing so, the researchers created an apparent cliff covered by a transparent pane (see **Figure 9**). In this setting, infants capable of crawling typically hesitated to cross the surface, despite encouragement from a parent on the other side of the cliff. The effect prevailed even when children were allowed to establish through touch that the surface was rigid and when they witnessed that a hard ball was able to bounce off the surface (Gibson and Schmuckler, 1989). Though it seems obvious that humans improve their ability to think of transparent barriers as impenetrable entities as they grow older, how this improvement takes place and to what extent is less clear. What seems evident is that even adults occasionally question the rigidity and durability of transparent barriers as they explore their environment.

This phenomenon is nicely illustrated by widespread responses toward glass walkways as erected over natural cliffs (e.g., in the Grand Canyon^6^; in the Tianmen Mountain^7^) or on top of modern buildings (e.g., the Transparent Observatory at the Oriental Pearl Radio and TV tower^8^). When entering such walkways, many visitors hesitate to step forward, despite witnessing other people standing or moving around on the same structure^9^ (Morse, 2011). Crucially, this hesitation tends to linger throughout a person’s initial steps and despite a contact-experience that signals the surface’s impenetrability. This phenomenon further confirms that in response to conflicting tactile and visual experiences humans are inclined to rely more strongly on the latter. Importantly, overcoming this overreliance on visual information in the presence of transparent barriers does not only seem to require extensive practice, but also consistent awareness of the problem at hand. People suffering from cognitive decline, such as Alzheimer patients, have been found to cope poorly with transparent environments. Their struggle to open glass doors (rather than trying to directly pass through them) or to look for alternative paths around transparent barriers suggests that they have lost the ability to overcome the visual illusion of penetrability caused by such structures (Passini et al., 2000).

## The Impact of Transparent Barriers on Social Behavior

Aside from investigating people’s visual, tactile, and spatial representations of transparent barriers in their environment, the barriers’ impact on people’s social functioning has attracted initial scientific attention (Procter, 1970). To understand the consequences of transparent barriers on social interactions more fully, the interplay between these structures and the types of social behavior they may foster and/or hinder must be considered (cf. Drew, 1971; Knapp et al., 2014; Patterson and Quadflieg, 2015). The current section focuses therefore on two types of social situations in which transparent barriers may impact social behavior: situations in which people are *separated* from each other by transparent barriers and situations in which people are *surrounded* by transparent barriers. Before addressing these two types of situations, the link between social interactions and their physical environments is considered more broadly.

Decades of social–psychological research suggest that people’s physical surroundings fundamentally shape social processes related to privacy, crowding, interpersonal involvement, and territoriality. Privacy is best understood as a dialectic process through which people strive for some momentary optimal level of contact with others (Altman, 1975). A person’s temporary need for privacy may be violated by crowding. Crowding refers to a negative affective response elicited by a high density of people in a specific location (Stokols, 1972). To regulate privacy under conditions of crowding, people may adjust their interpersonal involvement with others, that is, they may avoid close interpersonal distance, mutual gaze or touch, facial expressions of approachability, and so on (Patterson, 2011, 2013). Alternatively, people may try to own and/or control access to specific physical locations, thus showing territorial behavior (Altman, 1975; Bell et al., 2001; Brown, 2009; Scannell and Gifford, 2010). Social discomfort resulting from privacy violations, crowding, unwanted interpersonal involvement, and territorial intrusions is a common experience in everyday life. Its occurrence is particularly likely when strangers share a common presence, as is the case in many public settings, such as restaurants, waiting rooms, elevators, public transportation, open plan offices, or checkout/ATM lines (e.g., Camperio and Malaman, 2002; Manzo, 2005; Evans and Wener, 2007; Li and Li, 2007). In managing negative experiences around privacy, crowding, interpersonal involvement, and territoriality people not only adjust their own non-verbal behavior relative to others, but also use and manipulate elements in their physical environment.

Barriers, both permanent (e.g., walls, columns, partitions) and/or movable (e.g., furniture or plants), play a pivotal role in the regulation of social interactions between strangers (Levitt and Weber, 1989; Manzo, 2005; Robson, 2008). These barriers, collectively termed *anchors* (Robson, 2002), can limit spatial access to a person and provide temporary screening from the sight, sound, or proximity of others (Robson, 2008). What remains uncertain is whether people also treat transparent barriers as architectural elements with *anchoring* qualities. Transparent barriers form unique kinds of barriers, given that they separate space physically, but not visually. Toddlers as young as 14 months of age understand that, unlike an opaque barrier, a transparent barrier fails to block another person’s line of sight (Dunphy-Lelii and Wellman, 2004). Thus, both children and adults assume that a physical separation through a transparent barrier does not interfere with the transmission of visual information. Given that the exchange of visual information remains unimpaired, to which extent does a separation of strangers by a transparent barrier impact social behavior?

**FIGURE 10:**
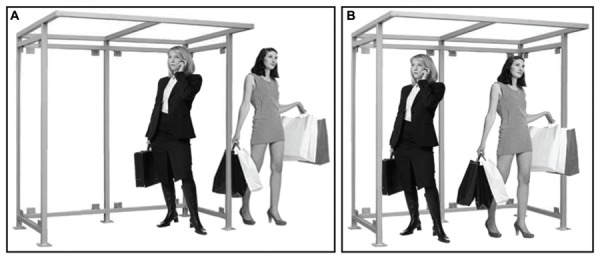
**Schematic depiction of two people waiting at a bus stop illustrating an uninvestigated question of social relevance: Would the close presence of a stranger be more comfortable when occurring **(A)** across a transparent barrier or **(B)** without such a barrier? [Images of humans were downloaded from www.shutterstock.com and are reproduced in this manuscript in adherence with the company’s standard license terms of service (http://www.shutterstock.com/licensing.mhtml)]**.

Though architects have long claimed that the visual and acoustic permeability of barriers affects social encounters (Zeisel, 1981), empirical investigations on this topic remain rare. In consequence, the impact of transparent barriers on social behavior is poorly understood. Consider, for instance, the observation that most people try to maintain a minimal interpersonal distance from unfamiliar others (Hall, 1959; Sommer, 1959). When a stranger initiates an inappropriately close approach, arousal, and discomfort result (Sommer, 1959; McBride et al., 1965; Middlemist et al., 1976; Patterson, 1976; Hayduk, 1983). Interpersonal distance violations can precipitate flight from a setting or, at least, compensatory non-verbal behavior, such as turning away or avoiding gaze to reduce the effect of a stranger’s close presence (Sommer, 1969; Patterson et al., 1971; Patterson, 1973; Konečni et al., 1975). It remains unclear, however, whether people’s need for controlling their involvement with others depends on the presence or absence of transparent barriers between them. On one hand, it could be argued that transparent barriers should not alter interpersonal distance preferences because visual information from others remains unobstructed. Alternatively, because transparent barriers interfere with tactile, auditory, and olfactory input (Crusco and Wetzel, 1984; Haans and IJsselsteijn, 2006), responses to the perceived proximity of others may change in their presence. By enabling the visual processing of others while preventing a physical interaction, transparent barriers may alter how we respond to the proximity of strangers (see **Figure 10**).

Similarly unresolved is the issue of how transparent barriers affect tacit norms of looking behavior. Establishing eye contact with another person frequently serves as a signal to initiate (verbal or non-verbal) communication (Kleinke, 1986). Thus, when such communication is not sought, people tend to respect others’ privacy by not looking at each other (Argyle and Dean, 1965; Laidlaw et al., 2011). But how are looking norms affected by the presence of transparent barriers? Are people inclined to stare longer at others through transparent barriers, knowing that under these conditions they can gather social information without the obligation to engage in any further exchange? Equally important is the questions of whether targets of prolonged looks may be more comfortable with attracting someone’s gaze through a transparent barrier than when no barrier is present. Because neither a spatial intrusion, nor a verbal approach is likely to follow under such conditions, there may be less discomfort resulting from being looked at in the presence of a transparent barrier compared to a no barrier arrangement.

Social interactions may also change their course depending on whether they are taking place in a space surrounded by transparent barriers. Experimental studies have demonstrated, for instance, that close spatial proximity to strangers produces less discomfort in open than in confined spaces (Cochran et al., 1984). Along similar lines, encounters with members of social outgroups (e.g., people perceived as having a different racial background than a perceiver) elicit associations related to a fight when they occur in a small booth, but to flight when they occur in an open field (Cesario et al., 2010). In other words, confined spaces defined by non-transparent barriers seem to encourage aggression, rather than withdrawal, during stressful social encounters. Whether people consider spaces largely defined by transparent barriers as confined or open, however, remains an issue of debate. Given that transparent barriers can be as impenetrable as their non-transparent counterparts, they can clearly be understood as physically confining. Their visual permeability, however, may reduce a person’s sense of confinement (Stamps, 2010). As a result, the same density level may reduce feelings of crowding in spaces largely defined by transparent barriers than in spaces defined by non-transparent barriers. The effect of perceived spaciousness, in turn, may translate into a decreased readiness to aggress and an enhanced willingness to consider withdrawal during perceived social threat. In contrast, feelings of crowding may occur even in low density spaces if there is a high-density environment on the other side of a transparent barrier. In other words, humans may not be affected by perceived spaciousness per se, but by the actual content of the views through their surrounding transparent barriers (Kaplan, 2001). Thus, a person’s readiness to aggress against social threats encountered indoors may get reduced or amplified, depending on whether transparent barriers afford stress-reducing or stress-inducing views (e.g., exposure to nature versus built environments; Kahn et al., 2008; Bratman et al., 2015). With increased globalization and migration, many contemporary societies are characterized by frequent encounters between strangers from different ethnic, cultural, and/or religious backgrounds. A large body of work indicates that such encounters often elicit mutual discomfort and anxiety (Stephan and Stephan, 1985; Smith and Mackie, 2010). If design features could enhance the safety and comfort of such interactions, architects should consider their regulatory impact when designing public spaces that welcome human diversity.

The lack of empirical data addressing the above issues is particularly unfortunate because many closely related questions of importance remain equally unaddressed. For example, can beneficial consequences of social proximity, such as the inhibition of stress hormones in the presence of social ingroup members, occur across transparent barriers (cf. Millidine et al., 2009; Beckes and Coan, 2011)? Are spaces bounded by transparent barriers sufficient to fulfill privacy needs? To what degree can transparent barriers define a territory that is recognized by others? Finally, can spaces defined by transparent barriers influence social interactions via changes in environmental features, such as lighting conditions and the level of visual stimulation? Initial evidence suggests, for instance, that in work settings people prefer rooms with sunlight and outdoor views (Wang and Boubekri, 2010; Aries et al., 2015). But can such preferences shape the course and quality of social interactions? Changes in light conditions over the course of a day can certainly entrain circadian rhythms and modulate physiological states, such as a person’s endocrine levels and heart rate (Edelstein et al., 2007). Further evidence suggests that lighting conditions around transparent barriers may even impact people’s social behavior. Brighter rooms, for instance, seem to facilitate social inhibition (Hirsh et al., 2011), an effect that can reduce anti-social behavior (such as aggression or dishonesty, see Page and Moss, 1976; Prentice-Dunn and Rogers, 1980; Zhong et al., 2010) but also prosocial behavior (such as the willingness to collaborate, see Steidle et al., 2013). Most importantly, these rare examples of experimental research show that the effects of transparent barriers (beyond corresponding changes in lighting conditions) on social exchange deserve empirical attention in order to understand these barriers’ impact on our everyday life.

## Practical Implications

A pivotal methodology, so-called evidence-based design, promotes the integration of traditional, predominantly intuition-driven architectural design with evidence-based decision-making (Rosswurm and Larrabee, 1999; Brown and Ecoff, 2011). The approach involves systematically tracking, comparing, and evaluating the consequences of architectural decisions on human health and wellbeing, so that the obtained findings can be applied to the design of new buildings (Lohr, 2004). The idea of evidence-based design is particularly relevant when it comes to the use of transparent barriers. Not only are such barriers unique in their dual nature (i.e., they are simultaneously absent and present), but they also form a novel type of environmental structure from an evolutionary point of view (Brzezicki, 2013a). In other words, the requirement to respond to and navigate transparent barriers has emerged only recently in human phylogeny. So what goals do architects typically have when using transparent design features and what is the evidence that these goals are met?

As frequently discussed in architectural circles “the quality, or state of being transparent is both a material condition […and…] an intellectual imperative” (Rowe and Slutzky, 1963). This twofold meaning of transparency has turned glass architecture into a marketing tool used to symbolize accessibility (e.g., in financial or governmental institutions; Whiteley, 2003) and democratic information exchange (Barnstone, 2005). Indeed, initial observations suggest that openness and transparency in workplace settings can facilitate productivity and innovation by enhancing the exchange of knowledge and skills between individuals (e.g., Hascher et al., 2002). At the same time, however, employees’ preferences and demands for privacy and defensible territories may interfere with architectural ideals of transparency (Kim and de Dear, 2013). Anecdotal evidence reveals that in modern buildings plants, posters, and other non-transparent items are often strategically placed to cover facades and interior walls made of glass. The well-known artist Wassily Kandinski, for instance, was once observed to cover a transparent glass wall of a Bauhaus building in white paint in order to avoid being constantly looked at by passersby (cf. Whiteley, 2003). These user-driven changes to built environments frequently signal contrasting preferences between architects, who aim to dematerialize spatial boundaries by using transparent glass, and building occupants who strive to re-establish them.

Differential preferences for transparency between architects and users may contribute to lethargy and difficulties concentrating as discussed in the context of the so-called *sick building syndrome* (Apter et al., 1994; Sahlberg, 2012). Although the potential contribution of transparent boundaries to this syndrome has yet to be studied, their role deserves particular scientific attention due to the profound perceptual, behavioral, and social repercussions as described in this article. At the same time, the use of transparent materials also merits consideration in the context of *healing architecture*. In contrast to the sick building syndrome, the concept of healing architecture refers to design features that promote human health and wellbeing. The term is usually applied in the context of healthcare buildings, where it denotes the capacity of architectural design to promote healing processes in the people it accommodates. A seminal article in this field “View through a window may influence recovery from surgery” was published in *Science* magazine (Ulrich, 1984). The authors analyzed outcomes of patients with gall bladder surgery. After undergoing the procedure, patients were accommodated in rooms on the second and third floors of a three-story wing of a hospital building. Windows of the patient rooms on one side of the wing looked out on either a grove of trees or on a brown brick wall. The results showed faster, less painful recovery for surgical patients with a windowed view to a natural setting than for those with a view of a brick wall. The authors noted, however, that many physical attributes in addition to view itself, such as the quality of light may have influenced the obtained results. Despite its ambiguity, this landmark study inspired further studies to investigate the relationship between architectural design and positive health outcomes (for a review see Lawson, 2010). Though it is generally agreed upon that environmental modifications can hardly ensure recovery from injury or disease, healthcare professionals increasingly recognize that architectural design can act as therapeutic assets by affecting occupants’ mood and social interaction patterns (Sommer, 1974; Kahn et al., 2008; Sternberg, 2009).

Building on this work, architects to date must strive to understand in which kinds of environments transparent barriers are likely to act as stressors or healers. In addition, they should explore the versatile cognitive and behavioral responses that humans adopt upon encountering and inhabiting transparent environments. A particular focus should lie on identifying human responses that arise specifically from adapting toward these rather novel environments. To systematically study the richness of human responses toward transparent barriers, the concept of *behavior settings* as originally introduced in ecological psychology may prove helpful (Barker, 1968). A behavior setting is a bounded geographical area in which human and environmental components interact in a coordinated fashion to facilitate an ordered series of events over a period of time (Wicker, 1979). Examples of a behavior setting include an office meeting, lunch at a restaurant, a church service, or a university lecture. Importantly, transparent barriers may have strikingly different consequences on human functioning across different behavior settings. A lecture room separated from a busy corridor by a transparent barrier, for instance, might interfere with students’ attention to the lecturer. In contrast, a transparent wall separating two administrative assistants working on collaborative tasks, but needing acoustic screening, may facilitate their effectiveness. Considering these examples, the decision to erect transparent barriers, either in order to replace non-transparent ones or to subdivide previously open spaces, should always entail evaluating potential changes in the series of events that characterize a specific setting.

Healing, or at least beneficial, effects of transparent barriers may be particularly likely in settings that require individuals to simultaneously connect with others, but also to protect their privacy. In this regard, open plan offices (i.e., offices that are not fully enclosed by internal walls) provide an interesting starting point for effective transparent barrier use. Open plan designs generally aim to optimize communication and information flow across individuals. Yet, their practical implementation is frequently accompanied by complaints about privacy violations (cf. De Croon et al., 2005; Kim and de Dear, 2013; De Been and Beijer, 2014). These complaints partially arise from intrusions caused by noise pollution from neighboring work stations (Lee and Brand, 2005; Veitch et al., 2007; Kaarlela-Tuomaala et al., 2009; Jahncke et al., 2011). Erecting transparent barriers between work stations may therefore allow architects to keep spaces visually connected, yet acoustically separated. In addition, placing workstations in close proximity to a window with an outside view may help employees cope with privacy challenges in open plan designs (Yildirim et al., 2007; Aries et al., 2010; Lottrup et al., 2015).

Finally, people not only select settings, but settings also select people (Wicker, 1979). That is, humans are rarely in a particular environment by chance. For example, behavioral scientists are likely to be found in university classrooms and research labs, but less frequently in corporate boardrooms or machine shops. The combined selection by individuals and settings increases the likelihood that people in a particular setting are more similar to one another than are people randomly sampled from a range of different settings. The process of structural constraints acting in concert with self- and setting-selection processes, social norms, and shared goals to limit behavioral options and to increase coordination in a given setting has been termed *synomorphy* (Wicker, 1979). The extent to which *synomorphic* mechanisms arise from environments with transparent barriers is another matter of speculation. It seems worthy of investigation, for instance, whether people with chronically low privacy needs, reduced impression management concerns, low territoriality claims, and/or claustrophobic tendencies experience greater satisfaction in surroundings with transparent barriers than people with contrasting needs and motives (e.g., people with high privacy needs and/or chronic fears of social evaluation by others). In a related manner, the use of glass features in architecture might be most welcome by occupants from cultures that are willing to publicize their everyday lives (Kükelhaus, 1973; Vera, 1989; Abel, 1997; Rieger-Jandl, 2006).

## Concluding Remarks

As interest in understanding and predicting how people respond to built environments begins to grow, the need for systematic research on the affective, cognitive, and behavioral responses to varied environments increases. The widespread, and occasionally undifferentiated, use of transparent barriers in modern construction poses a pivotal example of how architectural decisions could benefit from valid and reliable data in order to ensure a space’s functionality and user-friendliness. Through focusing on how transparency is experienced visually, haptically, and socially, the present paper integrated a number of seemingly disparate domains in a multidisciplinary manner. In doing so, it revealed the impact of transparent surfaces on various important aspects of human behavior. It was shown that seeing transparent barriers requires the detection and integration of several visual features, a circumstance posing a unique challenge to the visual system. Failure to detect transparent barriers, irrespective of their protective or restraining function, is common and can result in unintentional collisions. Further evidence suggests that such collisions are possible even upon the detection of a transparent barrier. That is, very young children and individuals suffering cognitive decline struggle with navigating transparent structures in their environments. These observations signal that the human mind has to actively construe the presence of a transparent entity, a process that requires experience with the material as well as the capacity to retrieve these experiences.

Furthermore, although plenty of evidence suggests that a cognitive understanding of transparent barriers as solid separators of space is acquired at an early developmental stage, even healthy adults occasionally treat transparent barriers as if they were *not there*. Both, tests of reflexive responding around such barriers as well as high-stake transactions with the material (e.g., requiring to pass a high cliff on a transparent walkway) reveal a human tendency to respond to their environments predominantly on the basis of incoming visual information. As a result, the unique property of transparent barriers – their visual penetrability – can override a person’s tactile experience and/or explicit knowledge that such barriers form solid separators of space. This tendency to consider transparent barriers as *not there* is also seen in studies on distance estimation. Several studies failed to detect spatial bias around glass barriers. This observed lack of bias may signal that transparent barriers are less likely to aid a perceiver’s ability to remember and navigate a space’s layout than opaque barriers, a possibility that requires further empirical examination. Similarly pressing is the question of how transparent barriers affect issues of privacy, crowding, territoriality, and interpersonal involvement. Most importantly, the existing work indicates that building transparent structures to serve complex human needs and goals requires a design process grounded in more than “intuition.” That is, the impact of transparent structures on human perception, cognition, and behavior needs to be systematically researched, combining the expertise of architects, designers, and behavioral scientists. The current review calls for such cross-disciplinary investigations as they form the stepping stones necessary for developing detailed recommendations and guidelines for the architectural use of transparent barriers.

## Conflict of Interest Statement

The authors declare that the research was conducted in the absence of any commercial or financial relationships that could be construed as a potential conflict of interest.

## Footnotes

1 It should be noted that the scholarship on transparent barriers summarized in this article was identified by searching two prominent academic databases (i.e., Google Scholar and Scopus) for the terms “glass architecture,” “transparency perception,” “transparent barrier perception,” “transparent barrier detection,” “transparent barrier AND distance,” “transparent barrier AND privacy,” and “transparent barrier AND space.” Once relevant work was identified, its bibliography and citations were searched for further material of interest. Material was included in this review if its content (a) referred to human cognition and/or behavior, (b) elucidated architectural, perceptual, navigational, or social aspects of transparent barrier use, and (c) was representative of a major line of research on the topic (i.e., not every single paper on the role of reflections in transparency detection was included, but rather typical exemplars). Finally, reviewer suggestions were incorporated to optimize the manuscript.

2 http://www.chncpa.org/ens/

3 www.gardenofwales.org.uk.

4 www.farnsworthhouse.org

5 http://theglasshouse.org/.

6 http://www.grandcanyonwest.com/skywalktour.php

7 http://www.tourismchina.org

8 http://www.orientalpearltower.com/en/index.html

9 https://www.youtube.com/watch?v$=$Z8WeJD2lc30
